# Sinonasal NUT carcinoma: A retrospective case series from a single institution

**DOI:** 10.3389/fsurg.2023.1098704

**Published:** 2023-03-01

**Authors:** Lei Wang, Zhenzhen Zhu, Weiqing Wang, Yang Zha, Xiaowei Wang, Aodeng Surita, Yuzhuo Liu, Wei Lv

**Affiliations:** Department of Otolaryngology-Head and Neck Surgery, Peking Union Medical College Hospital, Chinese Academy of Medical Sciences, Peking Union Medical College, Beijing, China

**Keywords:** NUTM1 protein human, paranasal sinuses, nasal cavity, prognosis, molecular targeted therapy

## Abstract

**Purpose:**

Nuclear protein in testis (NUT) carcinoma is a rare, aggressive tumor defined by the presence of *NUT* gene rearrangement. The aim of this study was to describe the clinical, radiologic, and biological features of sinonasal NUT carcinoma.

**Methods:**

We retrospectively investigated NUT expression with clinicopathologic features in 145 cases with sinonasal malignancies diagnosed from January 2017 to December 2021 and reviewed the reported cases.

**Results:**

Three (3/145, 2.07%) cases showed strong nuclear expression for NUT immunohistochemical, including one male and two females with ages from 37 to 57 years (mean, 45.33 years). All three cases involved the nasal cavity and sinuses; one of them involved the orbit and intracranial area. Histologically, all subjects showed poorly differentiated, small round cell morphology with distinct nuclei. All patients received surgery and chemoradiotherapy. One patient died of the disease 13 months after diagnosis, and two survived 12 and 15 months, respectively, without evidence of tumor recurrence. 51 cases of sinonasal NUT carcinoma (mean age 40.96 years) have been described to date. Among them, 28 are male, and 23 are female. Most cases expressed p63, AE1/AE3, as well as p40.

**Conclusion:**

NUT carcinoma is a rare and aggressive disease with a poor prognosis. It is crucial to perform *NUT* rearrangement-related tests for differential diagnosis of poorly differentiated/undifferentiated tumors in the nasal cavity and sinuses.

## 1. Introduction

NUT (nuclear protein in testis) carcinoma is a type of poorly differentiated or undifferentiated malignancy defined by the rearrangement of the *nuclear protein in testis (NUT)* gene (also known as *NUTM1*) (1, 2). The first case with chromosomal translocation *t*(15;19) involving the thymus was reported in 1991 (3). Since most cases were found in the midline of the body, such as the thorax or head and neck, it was first called “NUT midline carcinoma.” Afterward, many cases have been diagnosed arising outside the midline (4, 5).

Sinonasal NUT carcinoma is relatively rare, and the actual incidence is unknown due to the lack of comprehensive analysis of a large number of tumors as well as the underdiagnosed (6, 7). For instance, Lee et al. analyzed 362 cases of poorly differentiated or undifferentiated carcinoma of the head and neck, four (1.1%) of which were sinonasal NUT carcinoma (8). And of 151 cases of primary sinonasal carcinoma diagnosed at Johns Hopkins Hospital, only three were NUT positive (9).

In 2003, French et al. identified the fusion gene *BRD-NUT* in NUT carcinoma, which can encode a chimeric protein blocking differentiation and maintain cells in a highly proliferative, poorly differentiated state (10–12). Most NUT carcinoma cases harbor a reciprocal translocation between the *NUT* gene on chromosome 15q14 and bromodomain and extraterminal motif (BET) family genes *bromodomain 4 (BRD4)* on chr19p131 (10). In addition to *BRD4*, *NUT* can also be fused to *BRD3*, *NSD3*, *ZNF532*, and *ZNF592* (5, 11, 13).

The prognosis for this tumor is comparatively poor, with median overall survival (OS) ranging from 6.5 to 9.7 months, according to different studies (4, 7, 14, 15). Most patients with NUT carcinoma will die from rapid disease progression because of early metastasis to local and distant sites (7). However, in some cohort studies, patients with head and neck NUT carcinoma had a slightly better prognosis than patients with thoracic NUT carcinoma (4). NUT carcinoma affects males and females equally, and though it can affect people of any age (range 0.1–80 years), the median age is in teens and young adults (median age 16–23.6 years) (4, 15).

At present, there are no treatment guidelines for NUT carcinoma. For head and neck NUT carcinoma, aggressive primary surgical resection (with or without postoperative chemoradiation or radiation therapy) is associated with significantly improved survival. Chemotherapy or radiotherapy alone is often not sufficient (5, 7). Several promising classes of drugs, including BET inhibitors (BETi) and histone deacetylase inhibitors (HDACi), have emerged as candidates for treatment (1, 13). Therefore, making an accurate diagnosis is essential for the choice of treatment.

Morphologically, NUT carcinomas present nested and sheet-like monomorphic, undifferentiated round oval cells with a small to moderate amount of cytoplasm and frequent cell division with necrosis. The chromatin is typically vesicular. Occasionally, it appears abrupt differentiation of squamous cells or keratinization. Although infiltrating lymphocytes are occasionally seen, a more common finding is the presence of infiltrating neutrophils (5, 9). In the sinonasal tract, the appearance of NUT carcinoma overlaps with those of other poorly differentiated neoplasms or small round blue cell tumors, including sinonasal undifferentiated carcinoma (SNUC), Ewing sarcoma/primitive neuroectodermal tumors (PNET), Epstein-Barr virus (EBV)-associated lymphoepithelial carcinoma, lymphoma/leukemia, olfactory neuroblastoma, small cell neuroendocrine carcinoma, melanomas, rhabdomyosarcoma and the recently described SMARCB1(INI1)-deficient sinonasal carcinoma (5, 16–19). The accurate diagnosis of sinonasal NUT carcinoma is difficult without ancillary tests.

The application of NUT rabbit monoclonal antibody (clone C52B1, Cell Signaling Technology) has greatly improved the diagnosis rate in recent years (5, 20). In addition to immunohistochemistry (IHC), fluorescence *in situ* hybridization (FISH) using *NUT* split-apart probes is a sensitive method for detecting *NUT* rearrangements (5).

In 2017, NUT carcinoma was added to the 4th edition of the World Health Organization (WHO) classification of sinonasal tumors for the first time (21). However, the lack of reliable morphologic features, its rarity, and the lack of awareness contribute to the underdiagnosis of NUT carcinoma (5). In this study, we retrospectively reported the clinical characteristic, histological appearance, treatment, and outcome of patients with sinonasal NUT carcinoma in order to raise clinicians’ awareness of this disease.

## 2. Materials and methods

### 2.1. Patient selection and clinical review

A total of 145 patients with sinonasal malignancies treated at Peking Union Medical College Hospital from January 2017 to December 2021 were reviewed retrospectively. Three of them showed strong positive for NUT IHC, and one was weakly positive. Further clinical histological and immunohistochemical reviews and FISH were performed on all NUT IHC positive cases. All pathological diagnoses were confirmed by experienced pathologists. Criteria for analysis included the description of the population, initial clinical and radiologic presentation, pathological features, treatment administered, and outcome. Tumor staging was performed using the 8th edition of the American Joint Committee on Cancer (AJCC) TNM staging system. This study was approved by the Ethics Committee of Peking Union Medical College Hospital, and the requirement of informed consent was waived.

### 2.2. Histology, IHC

Hematoxylin-eosin (HE) stained sections were assessed for cell morphology, growth pattern, presence or absence of squamous differentiation, and necrosis. IHC for NUT was performed on formalin-fixed paraffin-embedded tumor sections, using the rabbit monoclonal primary antibody against NUT (Cell Signaling Technologies, 3625) in a dilution of 1:50. Cases with diffuse (>50%) strong, speckled nuclear staining were considered as positive. IHC for p63 (Abcam, ab124762, 1:5,000 dilution) and PD-L1 (Proteintech, 66248, 1:5,000 dilution) were performed according to standard procedures. IHC slides were observed using a microscope (Leica DM6 B, Wetzlar, Germany).

### 2.3. FISH

FISH analysis of NUT IHC positive cases was performed using *NUT* break-apart probes (Anbiping, Guangzhou, China). FISH slides were observed using a microscope (Leica DM6 B, Wetzlar, Germany) under a ×100 objective. Red fluorescence (R) labels the *5'NUT* (15q14) probe, and green fluorescence (G) labels the *3'NUT* probe. The normal signal pattern is shown as two red-green fluorescence fusions (2F), and the typical positive signal pattern is 1G1R1F. A total of 200 tumor cells were counted. If more than 15% contained *NUT* splitting signals, they were considered positive for FISH.

## 3. Results

### 3.1. Clinical data and radiologic characteristics

The 145 cases of sinonasal malignancies we retrieved exhibited a wide variety of pathological types (e.g., olfactory neuroblastoma, adenoid cystic carcinoma, sarcoma, etc.). Of these, 5 were undifferentiated and 30 were poorly differentiated. A total of three cases (3/145, 2.07%) were strongly positive for NUT IHC, and one was weakly positive (eventually diagnosed as sinonasal poorly differentiated squamous carcinoma). The age at diagnosis ranged from 37 to 57 years, with a mean age of 45.33 years. The male-to-female ratio was 1–2. All three cases involved the nasal cavity and sinuses, one involved the orbit and intracranial region, and two had cervical lymph node metastases. They were treated with radical surgical resection and all obtained negative margins at the initial surgery. In addition, all three underwent postoperative radiotherapy. Two of them received chemotherapy. Prognostically, the patient with T4bN2M0 recurred 9 months after initial surgery and died 13 months after diagnosis due to intracranial recurrence/metastasis. The other two patients showed no signs of recurrence at the end of follow-up, which was 15 and 12 months, respectively. The clinical data of NUT carcinoma are shown in Table 1.

**Table 1:** Clinical characteristics of patients with sinonasal NUT carcinoma.

Case	Age/Sex	Tumor location	Symptom	Stage	Treatment	Outcome	Follow-up (months)
1	37/F	Left frontal and ethmoid sinuses; nasal cavity; orbit; anterior skull base	Eye and frontal bulge, vision loss, epiphora, head and face pain, nasal obstruction, nasal discharge stained with blood	T4bN2M0	S + CRT	DOD	13
2	42/M	Right ethmoid and maxillary sinuses; nasal cavity; nasopharynx	Nasal obstruction, rhinorrhea, dorsum nasi swelling, epiphora	T3N1M0	S + RT	NETR, lost	15
3	57/F	Left maxillary and ethmoid sinuses; nasal cavity	Left maxillofacial pain, nasal obstruction, rhinorrhea, nasal discharge stained with blood, epiphora	T3N0M0	S + CRT	NETR	12

CRT, chemoradiation therapy; DOD, died of disease; NETR, no evidence of tumor recurrence; RT, radiotherapy; S, surgery.

**Figure 1:**
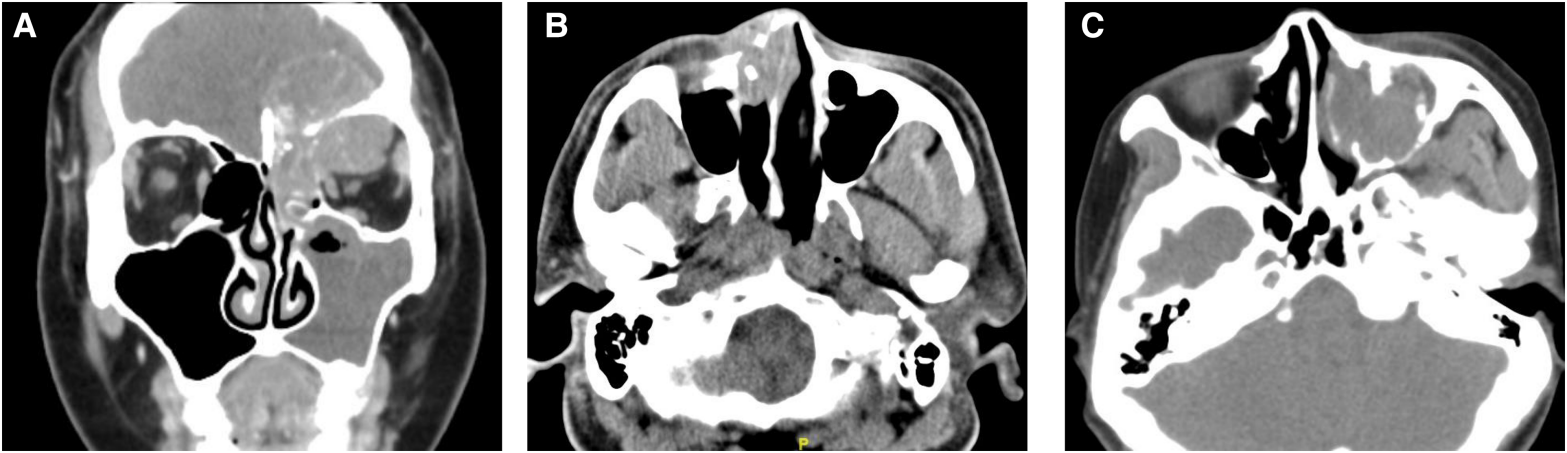
Computed tomography (CT) images of NUT carcinoma cases. (**A**) Coronal enhanced CT of case 1: the mass involved the left orbit, ethmoid sinus, and cranium. (**B**) Axial CT showed the tumor of case 2 located in the nasal cavity and destroyed the surrounding bone. (**C**) CT suggested a mass in the left maxillary sinus of case 3.

Case 1: A 37-year-old woman visited Ophthalmology due to a bulge above her left eye with vision loss for a month. Four years ago, she began to develop a nasal obstruction on the left with nasal discharge stained with blood. Computed tomography (CT) and magnetic resonance imaging (MRI) showed a mass in the left frontal sinus, involving the left orbit and ethmoid sinus, and the lesion extended to the cranium (Figure 1A). Follow-up positron emission tomography (PET)-CT suggested that cervical lymph node metastasis was possible. She had a history of smoking and no history of drinking. The patient received open surgery, followed by two cycles of chemotherapy (vincristine + ifosfamide + epirubicin) and radiotherapy (dose unknown). The tumor recurred 9 months after surgery. She underwent surgery again and 1.5 months later developed intracranial recurrence or metastasis. The patient died 13 months after the initial diagnosis.

Case 2: A 42-year-old male presented with right-sided nasal obstruction and rhinorrhea for 1 month. He also presented with ipsilateral dorsum nasi swelling and epiphora. The patient smoked and occasionally drank alcohol. CT (Figure 1B) and PET-CT suggested right maxillary sinus, ethmoid sinus, and nasal cavity masses with possible cervical lymph node metastasis. He underwent open surgery and radiotherapy (dose not known). No signs of tumor recurrence were seen 15 months after surgery, after which the patient was lost to follow-up.

Case 3: A 57-year-old woman visited our hospital for “left maxillofacial pain with nasal obstruction and rhinorrhea for 1 month”. CT (Figure 1C) suggested a mass in the left maxillary sinus, nasal cavity, and ethmoid sinus with multiple bone destruction. The patient had no history of alcohol or tobacco use. She underwent endoscopic surgery. Afterward, she received chemoradiotherapy at another hospital (protocol unknown). Postoperatively, she has been followed up for 12 months to date, and no tumor recurrence has been observed.

### 3.2. Pathological findings

**Figure 2:**
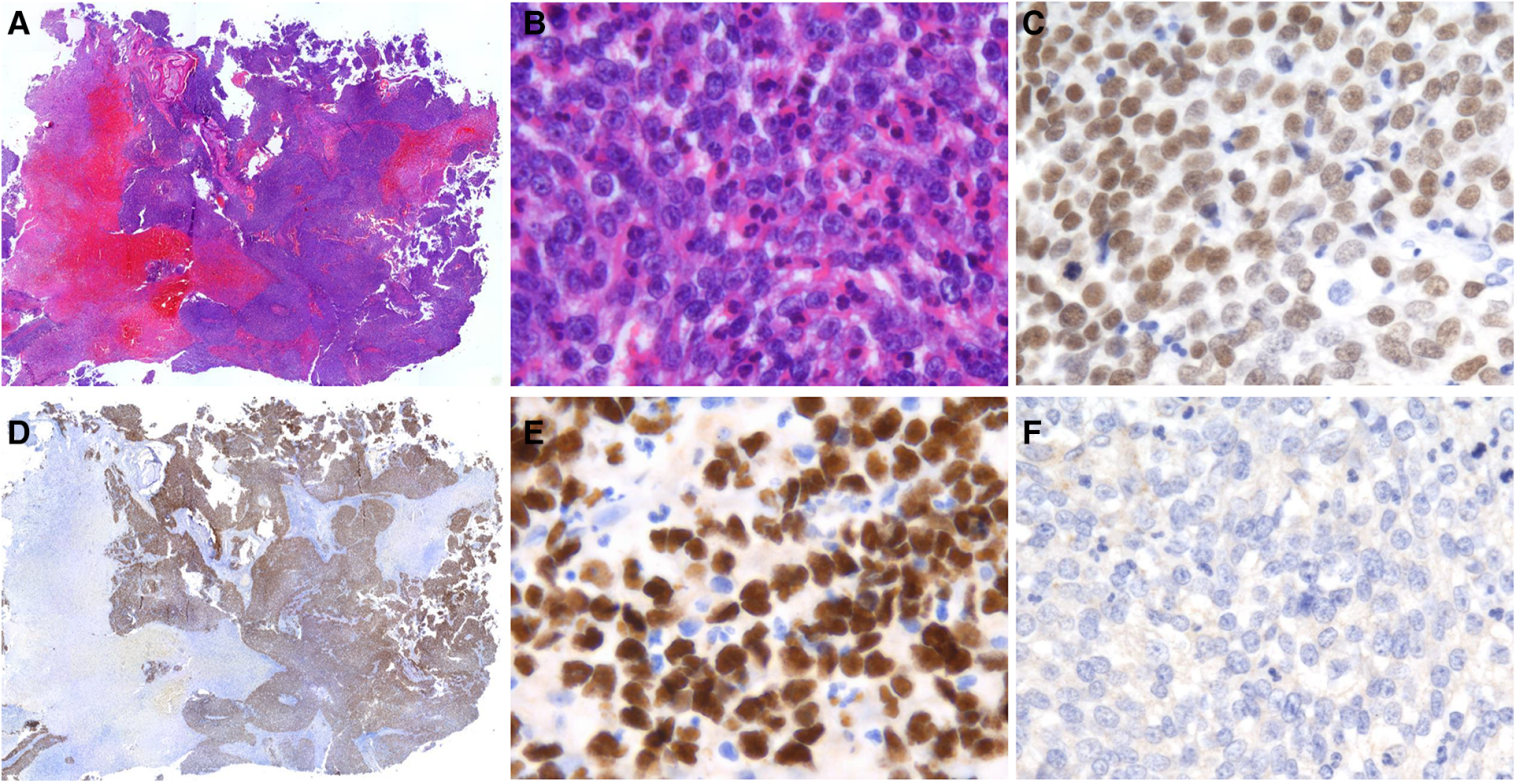
Histological and IHC findings of sinonasal NUT carcinoma. (**A**) HE staining showed that NUT carcinoma grew as sheets and nested of cells (Case 1, original magnification ×50). (**B**) The tumor was predominantly composed of small to middle-sized cells with scant cytoplasm. Marked infiltration of neutrophils was seen (Case 2, original magnification ×400). (**C**) Diffuse expression of p63 was observed in all cases (Case 1, original magnification ×400). (**D,E**) Positive nuclear NUT immunostaining was present (Case 1, original magnification ×50, ×400). (**F**) PD-L1 was negative in all cases (Case 1, original magnification ×400).

Histologically, all three cases presented with poorly differentiation. The tumor consisted of relatively homogeneous, small to medium-sized cells with sparse cytoplasm and deep-stained nuclei with prominent nucleoli. No abrupt squamous differentiation or keratinization was evident in the three cases we collected. Neutrophil infiltration was seen in all but case 3 (Figures 2A,B). NUT IHC was performed, showing speckled nuclear staining with *NUT* fusion characteristics (Figures 2D,E). Furthermore, IHC results showed that all tumor cells expressed p63 (Figure 2C) but not PD-L1 (Figure 2F). The results of other immunohistochemical parameters are detailed in Table 2.

**Table 2:** Pathologic features.

Case	HE	Positive tests	Negative tests	FISH
1	Poorly differentiation	NUT, AE1/AE3 (focal), p63, p40, Ki-67 (index 40–65%)	CD56 (NK-1), CD20, CD3, CgA, SYN, EBER-ISH	Positive
2	Poorly differentiation	NU, p63, Ki-67 (index 50%), p16 (focal), EGFR, p53, P40, NUT	CgA, p16, NSE, SYN, S100, EBER-ISH	Negative
3	Poorly differentiation	AE1/AE3, GFAP (focal), Ki-67 (index 80%), NUT	LCA, CD99, Desmin, EMA, Myoglobin, S100, Vimentin, NSE, HMB45, STAT6	*NUT* abnormity
*	Poorly differentiation	Myc, NUT (+/−), p63, p40, Ki-67 (index 40%), p53, AE1/AE3, Vimentin (focal)	CD117, SMA, SYN	Negative

EBV-ISH, Epstein-Barr virus (EBV) *in situ* hybridization.

* Case with NUT IHC weakly positive.

### 3.3. FISH results

**Figure 3:**
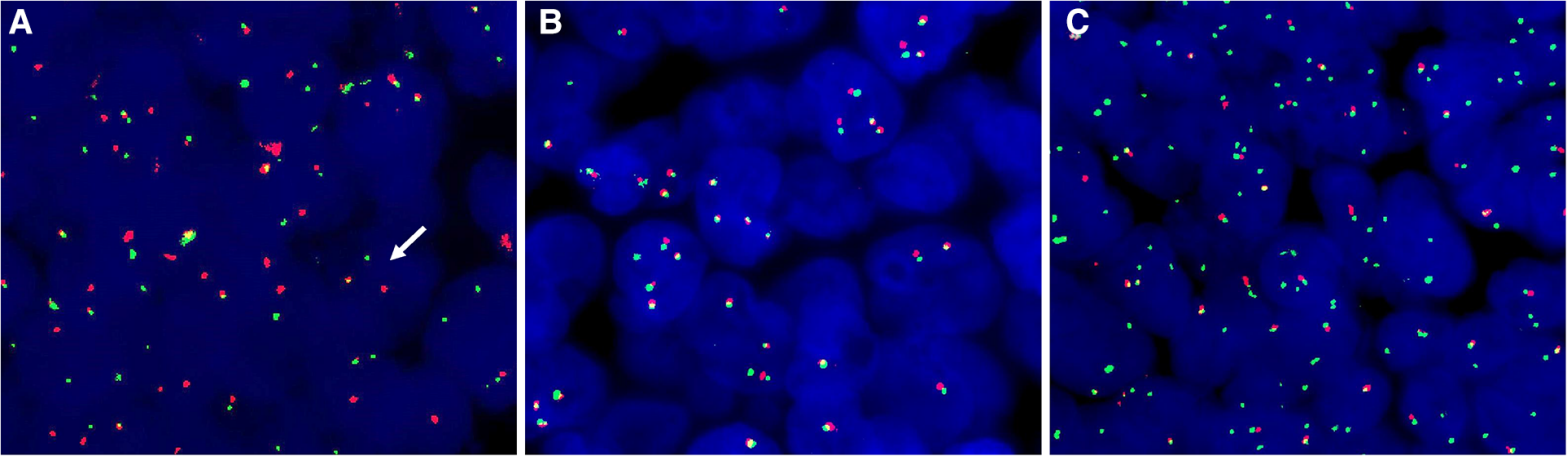
FISH with *NUT* break-apart probes. (**A**) Case 1 exhibited typical split signals. (**B**) Case 2 showed no splitting of *NUT* signals. (**C**) Additional *3'NUT* signals were present in case 3.

The three sinonasal NUT carcinoma cases exhibited different FISH results. Typical *NUT* break-apart was observed in 62.0% of the tumor cells in case 1 (Figure 3A). However, the percentage of typical splitting signals was meager (1G1R1F 1.5%) in case 2 (Figure 3B). Notably, case 3 lacked the typical *NUT* break signal. Nevertheless, the additional green signal was present in most tumor cells, demonstrating an atypical abnormality of the *NUT* gene (Figure 3C).

### 3.4. Pathological features of the NUT weakly positive case

**Figure 4:**
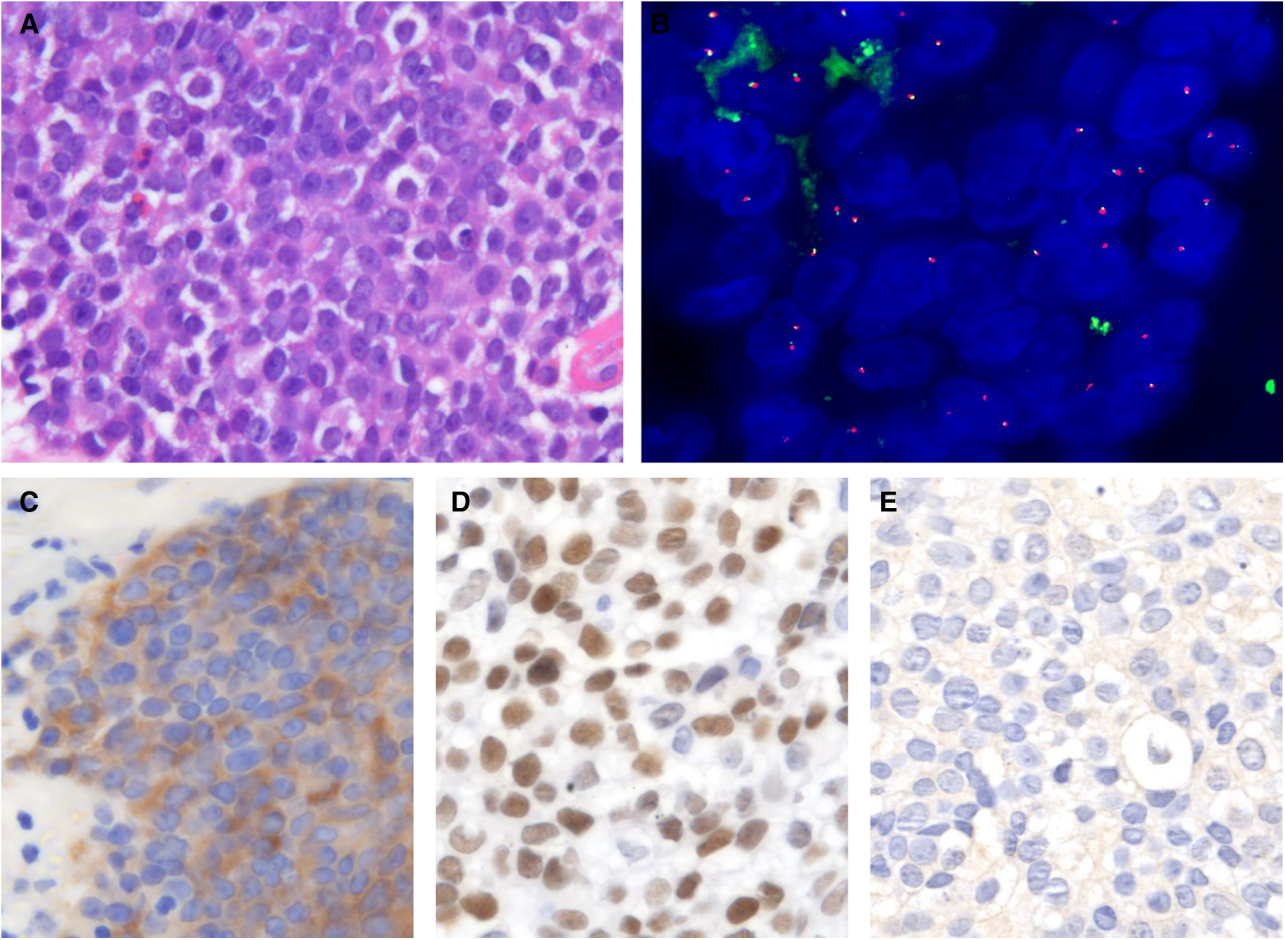
Histological and IHC features of the case with weakly NUT positive. (**A**) Tumor cell nuclei were large and round. (**B**) FISH assay was negative. (**C**) NUT positivity was seen in the cytoplasm but not the nucleus. (**D**) Diffuse p63 positivity is present. (**E**) Negative staining for PD-L1 (HE and IHC, original magnification ×400).

Morphologically, this case was very similar to the sinonasal NUT carcinoma: tumor also consisted of monomorphic cells with prominent nuclei, even with neutrophil infiltration. In addition, the tumor cells expressed p63 positively but not PD-L1. The apparent difference compared to NUT carcinoma was that the NUT-positive signal was present in the cytoplasm rather than the nucleus. Meanwhile, the FISH test was negative (Figure 4). Combined with other immunohistochemical tests, the case was finally diagnosed as poorly differentiated squamous carcinoma.

### 3.5. Characteristics of sinonasal NUT carcinoma with the review of literature

Approximately 51 cases of sinonasal NUT carcinoma have been described to date. It is important to note that some cases may have the potential to be reported repeatedly. For example, French et al. summarized the cases of NUT carcinoma at different years or from different perspectives in the study units (4, 7, 19). The age at diagnosis for these 51 cases ranged from 9 months to 67 years, with a mean of 40.96 years. Among them, 28 are male, and 23 are female, with a sex ratio of 1.22. Most cases expressed p63, AE1/AE3, as well as p40. In contrast, all relevant tests for EBV were negative. The details of each case are summarized in Table 3.

**Table 3:** Clinical and pathological features of sinonasal NUT carcinoma reported in the literature.

Year	Age (years)	Sex	Location	Metastasis	Therapy	Outcome	Follow-up (months)	Tests	Positive tests	Negative tests
2004 (2)	26	M	Sinonasal	Bone	CRT	Alive	67	IHC + FISH	CD34, NUT	PLAP
2009 (22)	31	M	Nasal cavity	LNs	CRT + S	DOD	10	/	/	/
	39	F	Nasal cavity, frontal sinus	LNs	CRT + S	DOD	7	/	/	/
2008 (23), 2010 (19)	31	M	Nasal cavity	/	/	/	/	IHC + FISH	p63, NUT	/
	39	F	Nasal cavity, frontal sinus	/	/	/	/	IHC + FISH	p63, NUT	/
	40	F	Nasal cavity and maxillary, frontal sinuses	/	/	/	/	IHC + FISH	p63, NUT	/
	47	M	Nasal cavity, ethmoid sinus	/	/	/	/	IHC + FISH	p63, NUT	/
2012 (9)	26	M	Paranasal sinus	LNs	S + CRT	DOD	/	TMA + IHC	NUT, AE1/AE3	/
	33	M	Paranasal sinus	Yes	S + CRT	DOD	/	TMA + IHC	NUT, AE1/AE3	/
	48	M	Paranasal sinus	Yes	S + CRT	DOD	/	TMA + IHC	NUT, AE1/AE3	/
	56	F	Sinonasl tract	LNs	/	/	/	IHC + FISH	NUT	/
	36	F	Sinonasl tract	Bone	/	/	/	IHC + FISH	NUT	/
2011 (22)	54	F	Right nasal dorsum	LN	CRT	DOD	7	CA + IHC + FISH	Vimentin, CAM 5.2, NUT	S100, CD99, NSE, CD56, SYN, myogenin, myo-D1, Desmin, CD45
2019 (1)	39	F	Paranasal sinus	/	/	/	/	FISH	CAM5.2, SYN	Pankeratin
	49	M	Frontal sinus	/	/	/	/	FISH	p63, CK5/6, CK7, p16	TTF1, SYN, CHR
	48	M	Ethmoid sinus	/	/	/	/	FISH	AE1/AE3, EMA	SYN, CHR, TTF1, GFAP
	67	F	Nasal, maxillary sinus	/	/	/	/	FISH	p63, SYN	CHR, S100, CD99, CK7, CK5/6, Desmin, CD34
2015 (24)	26	M	Left maxillary sinus, nasal cavity	/	S + CRT	DOD	18	IHC	NUT, CK AE1/3, CD99 (focal)	p63, CHR, Desmin, S100, EBV- ISH
2011 (17)	54	F	Left paranasal sinus	/	CRT + S	/	/	IHC + FISH	CK7, p63, NSE (+/−), NUT	EBV- ISH, S100, CD45, SYN, CHR-A, CK 20, CD34
2014, 2018 (16, 18)	18	F	Right nasal cavity and maxillary, ethmoidal sinuses	/	CRT	AWD	12	IHC + FISH + CA + RT-PCR	CD138 (foacl), AE1/3, EMA, p63, p40 (focal), Vimentin, NUT, Myc	S100, CD34, CD99, SYN, myogenin, PLAP, c-kit, hCG, CAM5.2, CK5/6, CGNA, SYN, CD56, Desmin, CD45RB, NKX2.2, TdT, p16, EBER-ISH
	56	F	Left nasal cavity, ethmoidal sinus, nasopharynx	Liver, lungs, pleura, spleen, adrenal glands, LNs, and bones	CRT	DOD	10	IHC + FISH + RT-PCR	CAM5.2, p63, p40 (focal), vimentin, NUT, Myc, CD56 (focal)	AE1/AE3, EMA, CK5/6, CD34, CGNA, SYN, S100, Desmin, CK45RB, NKX2.2, TdT, p16, EBER-ISH
	66	F	Frontal sinus	Liver and bones	CRT	DOD	13	IHC + FISH + RT-PCR	AE1/AE3 (focal), CAM5.2, EMA, p63, p40, Vimentin, NUT, Myc, CD34, CD56 (focal)	CK5/6, CGNA, SYN, S100, Desmin, CD45RB, NKX2.2, TdT, p16, EBER-ISH
	0.75	M	Bilateral nasal cavities, maxillary sinuses	Lungs, kidneys, bone, thyroid, liver, left adrenal gland, pancreas, right submandibular gland, and LNs	CRT	DOD	15	IHC + FISH + RT-PCR	AE1/AE3 (focal), p63, p40 (focal), Vimentin, NUT, Myc	CAM5.2, EMA, CK5/6, CD34, CGNA, SYN, CD56, S100, Desmin, CD45RB, NKX2.2, TdT, P16, EBER-ISH
2020 (8)	60	F	Right maxillary sinus	Yes	RT + S	DOD	12	IHC	Pan-CK, p63, p40, CD99 (focal weak), NUT	CD34, CD56, p16, EBV-ISH
	45	F	Left ethmoid sinus	No	S + CRT	NETR	36	IHC	Pan-CK, p63, p40, p16, NUT	CD34, CD56, EBV-ISH, CD99, HPV genotype PCR
	42	M	Right ethmoid sinus	Yes	CRT.	R	/	IHC	Pan-CK, p63, p40, CD99 (focal weak), NUT	CD34, CD56, p16, EBV-ISH
	29	M	Right ethmoid sinus	No	S + CT	/	/	IHC + FISH	Pan-CK (focal), p63, CD99 (focal), NUT	CD34, CD56
2015 (25)	14	F	Right nasal cavity, anterior ethmoid sinus	/	S + RT	DOD	3	IHC + FISH	NUT	/
2018 (26)	30	M	Left sinonasal	/	/	/	/	IHC	CK, NUT, p40, p16 (focal)	CD34
	31	F	Left nasal cavity and ethmoid, sphenoid, maxillary sinuses	/	CRT + S	DOD	2	IHC	NUT, p40, p16 (focal), CD34, p16 (focal)	/
	25	M	Right nasal cavity	/	/	/	/	/	CK, NUT, p40, p16 (focal)	SYN
	10	F	Left nasal cavity, left lacrimal sac	/	S + CRT	/	/	/	NUT, p16	
	30	F	Left nasal cavity, maxillary sinus, orbit	/	/	/	/	/	Pan-CK, p40, NUT, p16 (focal)	SYN, EBV-LMP
2017 (14)	20	M	Left ethmoid sinus, orbit	No	CRT + S	DOD	22	IHC + RT-PCR	AE1/AE3, CK, NUT	TTF1, Desmin, myogenin, CD45, CD34
2016 (27)	20	M	Sinonasal	/	S + CRT	DOD	22	FISH	AE1/AE3, CK14, CK5/6	SYN, CHR-A, CD56, S100
2017 (28)	53	M	Left nasal cavity	No	S + CRT	DOD	3	IHC + FISH	CK5/6, p16, p40, p63, NUT, CK20 (focal), SYN	CD34, S100
2018 (29)	49	M	Left nasal cavity and maxillary, ethmoid, frontal sinuses; right frontal sinus	Bone, LNs	CRT + S	DOD	9	IHC + CA	CD99, NUT, CAM5.2, S100 (focal)	AE1/AE3, CD3, CD20, CD56, SYN, Desmin, myoglobin
2021 (30)	56	F	Right nasal cavity	/	S + CRT	DOD	6	IHC	pankeratin, p16, p53, NUT	/
2020 (31)	44	M	/	/	/	/	/	IHC	NUT	/
2018 (32)	48	M	Left nasal cavity	/	S + CRT	DOD	/	MGT	Monokerati, p63, CD34, p16	S100, HMB45, leukocyte common antigen, CHR, SYN, EBV-ISH
2019 (33)	48	M	Left sphenoidal sinus	/	S + CRT	Alive	6	IHC + targeted RNA sequencing	NUT, AE1/3, CK5/6, p40, Ki67 (60%), SYN (weak), p16 (weak)	CHR-A, Desmin, S100, EBV-LMP
2021 (34)	39	M	Sinonasal	Lung	S + CRT + BETi	Alive	21	IHC + whole transcr-ipto-mic RNA sequencing	AE1/AE3, CK5, p40 (focal), NUT	TTF1, Napsin-A, SYN, CHR-A, smooth muscle actin, p16, S100, EBV-ISH
2015 (35)	29	F	Left maxillary sinus	LNs	S + CRT	/	/	/	p16 (partial)	EBER
2018 (36)	60	F	Nasal cavity	/	S + RT	DOD	3	IHC + FISH	NUT, CK5/6, CK7, p16, p40, p63, SMARCB1 (INI1), Vimentin, Ki-67 (95%)	CD34, CD56, CHR, S100, SYN
	65	M	Nasal cavity	/	S + RT	Alive	108	IHC + FISH	NUT, CK5/6, EMA, CK7, p16, p40, p63, SMARCB1 (INI1), Vimentin, ki-67 (95%)	CD34, CD56, CHR, S100, SYN
	46	M	Maxillary sinus	/	S + RT	DOD	8	IHC + FISH	NUT, CK5/6, EMA, p16, p40, p63, SMARCB1 (INI1), Vimentin, Ki-67 (95%)	CD34, CD56, CHR, S100, SYN
2022 (37)	60	F	Right sinonasal tract	/	S + RT	Alive	5	IHC + FISH	NUT, p63, CK5/6 (focal), p40 (focal), AE1/AE3	CHR-A, CD56, CD45RO, NKX2.2, p16, Vimentin, S100
2013 (38)	55	M	Sinonasal	Intracranial, orbit	RT	AWD	40	IHC + FISH	NUT, CK7, CK8, p63	/
	42	M	Sinonasal	Intracranial, orbit	RT + CRT	DOD	12	IHC + FISH	NUT, CK7, CK8, p63	/
	59	F	Nasal cavity	/	RT	AWD	12	IHC + FISH	NUT, CK7, CK8, p63	/
	50	M	Sinonasal	Intracranial	/	DOD	1	IHC + FISH	NUT, CK8, p63	/

AWD, alive with disease; CA, chromosome analysis; CHR, chromogranin; CK, cytokeratins; CRT, chemoradiation therapy; CT, chemotherapy; DOD, died of disease; EBV-ISH, Epstein-Barr virus (EBV) *in situ* hybridization; LNs, lymph nodes; MGT, molecular genetic testing; NA, not available; NETR, no evidence of tumor recurrence; NSE, neuron-specific enolase; Pan-CK, pancytokeratin; PLAP, placental alkaline phosphatase; R, recurrence; RT, radiotherapy; S, surgery; SYN, synaptophysin; TMA, tissue microarrays.

## 4. Discussion

The overall incidence of NUT carcinoma is very low. To better summarize and study the disease, in 2010, French et al. established the International NUT Midline Carcinoma Registry (INMCR) to perform analyses of clinical and pathologic data for natural history, therapeutic intervention, and outcome (7). From 1993 to 2014, 107 patients were collected in the INMCR, of which 48 (45%) were head and neck NUT carcinoma, with 57% originating in the nasal cavity. *BRD4-NUT* gene fusion was present in 86% of cases (7). Although sinonasal NUT carcinoma is relatively frequent (8), the number of reported cases is still rare, making it difficult to summarize the epidemiological features, optimal treatment options, and prognoses. Here we reported three cases from a single institution and summarized the previously reported 51 cases to improve the knowledge about the clinical, radiologic, and pathologic characteristics of this disease.

Histologically, NUT carcinoma is an undifferentiated or poorly differentiated cancer marked by the persistent expression of epithelial markers, such as whole pancytokeratins (AE1/AE3), CAM5.2, and EMA on IHC (14, 16). Besides, NUT carcinoma of the sinonasal tract can be positive for p63, p40, and CD34 (9, 16).

Interestingly, the case with weakly positive for NUT IHC in our study expressed Myc. Myc is expressed in a variety of tumors, including adenocarcinoma and lymphoma. Although Myc is not a specific marker for NUT carcinoma, evidence suggests that this oncogene plays a vital role in the disease (12, 16). NKX2.2 is a new sensitive marker to differentiate Ewing's sarcoma and olfactory neuroblastoma from other small round cell tumors (16). No NKX2.2-positive sinonasal NUT carcinoma cases have been reported to date. Previous studies have shown that NUT carcinoma lack expression of checkpoint immunotherapy markers (39). Similarly, none of our three cases expressed PD-L1 in tumor tissue.

There is no evidence that smoking or virus infection is associated with NUT carcinoma (5, 6). Consistent with previous reports, our cases were also negative for EBER-ISH. However, there were some sinonasal NUT carcinoma cases positive for p16 IHC in other studies (Table 3).

In addition to IHC, various assays can be used to identify *NUT* rearrangements, including FISH, reverse transcriptase polymerase chain reaction (RT-PCR), cytogenetics, and next-generation sequencing (NGS) (5). The three cases we reported exhibited different FISH results. However, FISH is not completely specific for diagnosing NUT carcinoma, and a negative result cannot be used as a definitive exclusion. Some unexpected cases of “cryptic” *BRD4-NUT* rearrangements strongly positive for NUT IHC were negative for standard FISH (20). For example, McLean-Holden et al. reported a case with negative FISH result diagnosed by IHC and RNA sequencing. The reason for false-negative FISH results in some NUT carcinoma cases is not entirely clear. However, it may be due to the fact that many *NUT* translocations are caused by chromosomal abnormalities, in which up to 30 rearrangements arise from a single catastrophic event resulting in a single oncogenic fusion (40). For the NUT carcinoma diagnosis, the sensitivity of FISH is 93%, and as a standard, IHC has a sensitivity of 87% and a specificity of 100% (20). More than 50% positive staining is considered diagnostic as NUT carcinoma according to the WHO tumor classification. Germ cell tumors such as seminoma, dysgerminoma, and embryonal carcinoma, or rare poorly differentiated carcinoma may also stain, but only focally (<10%) (5).

For poorly differentiated/undifferentiated malignant with relatively homogeneous morphology, it is necessary to perform NUT IHC assays for differential diagnosis (16). Accurate diagnosis is vital, not only because of the tumor's aggressiveness but also for detecting potential molecular targeted therapies. NUT cancer is unique in that epithelial cancers are usually characterized by multiple sequential mutations that can progress to carcinogenesis through a multistep pathway. Translocation-associated fusion oncoproteins are commonly found in hematopoietic and mesenchymal malignancies (11).

NUT is a protein with largely unknown functions, shuttling between the nucleus and cytoplasm (1). Under normal conditions, the *NUT* promoter is active only in adult testis and ciliary ganglia. Thus, only one type of the fusion genes is expressed, such as *BRD-NUT* (where the *BRD4* promoter and bromodomains drive aberrant *NUT* expression and chromatin binding), but not *NUT-BRD* (18, 41). The BET family of proteins consists of two tandem bromodomains (BD) and an extra-terminal structural domain (ET) (5). BRD2, BRD3, BRD4, and BRDT, these BETs are highly homologous (5). Normally, the function of BRD4 is to facilitate transcriptional elongation through the recruitment of CDK9/Cyclin T1 heterodimer (P-TEFb) (42). NUT is trapped in the nucleus when fused to BRD4 or BRD3. This is due to the acetylated lysine residues bound to and localized on the histone by the bromodomain protein. When NUT protein binds to histone acetyltransferase p300, p300 is isolated to the site of the BRD4/3-NUT complex, leading to local hyperacetylation of the histone (1). *In vitro* studies have shown that NUT fusion proteins drive tumor growth and block differentiation through aberrant histone acetylation depending on the targeting of *Myc* and *TP63* genes by BRD bromodomains (12). In addition to NUT carcinoma, other types of tumors may also have *NUT* gene rearrangements, such as sarcoma (1).

BET inhibitor drugs are acetylated histone analogs that competitively inhibit the binding of fusion products such as BRD4-NUT, and clinical trials have demonstrated efficacy in the treatment of NUT carcinoma. HDACi can promote overall histone acetylation and facilitate differentiation to the squamous phenotype, of which clinical results have also been seen (40). Nevertheless, approximately 1/3 of *NUT* rearranged tumors are so-called “*NUT* variants,” defined as cases in which *NUT* is fused to *non-BRD* genes, some of which do not encode or interact with bromodomain-containing proteins. This increases the likelihood that some cases will not respond to BETi therapy (1). Moreover, since BRD4 is expressed in most tissues, toxicity (most commonly thrombocytopenia) also limits the efficacy of BETi in the treatment (5).

Despite the availability of targeted drugs, the overall prognosis of NUT carcinoma remains poor. In a recent review of NUT carcinoma, Chau et al. reviewed 141 cases reported by the INMCR. Of these patients, only 16 survived at least 3 years, 6 survived at least 5 years, and only 1 survived at least 10 years (15). A few exceptional cases had more prolonged survival after diagnosis, ranging from 35 to 144 months (40).

In the present study, we sought to explore the pathological features and clinical manifestations of NUT carcinoma in the sinonasal tract. For this purpose, we retrospectively analyzed all of the sinonasal tumors in our hospital. Out of 145 cases of sinonasal tumors, a total of three cases were diagnosed as NUT carcinoma. It is important to note that the proportion of adolescent patients is lower than adult in our hospital. This could be the reason for the higher mean age of disease.

## 5. Conclusion

Sinonasal NUT carcinoma is a rare disease with aggressive behavior and a poor prognosis. Tests for *NUT* rearrangement should be performed in all suspicious cases, especially in the paranasal sinuses and nasal cavity.

## Data Availability

The raw data supporting the conclusions of this article will be made available by the authors, without undue reservation.
